# *INPP5D* rs35349669 polymorphism with late-onset Alzheimer's disease: A replication study and meta-analysis

**DOI:** 10.18632/oncotarget.12648

**Published:** 2016-10-13

**Authors:** Hua Jing, Jun-Xia Zhu, Hui-Fu Wang, Wei Zhang, Zhan-Jie Zheng, Ling-Li Kong, Chen-Chen Tan, Zi-Xuan Wang, Lin Tan, Lan Tan

**Affiliations:** ^1^ Department of Neurology, Qingdao Municipal Hospital, School of Medicine, Qingdao University, Qingdao, PR China; ^2^ Department of Emergency, Qingdao Municipal Hospital, School of Medicine, Qingdao University, Qingdao, PR China; ^3^ Department of Geriatric, Qingdao Mental Health Center, Qingdao, PR China; ^4^ College of Medicine and Pharmaceutics, Ocean University of China, Qingdao, PR China

**Keywords:** Alzheimer's disease, INPP5D, rs35349669, association study, meta-analysis, Gerotarget

## Abstract

Inositol polyphosphate-5-phosphatase (*INPP5D*) was reported to be associated with Alzheimer's disease (AD) through modulating the inflammatory process and immune response. A recent genome-wide association study discovered a new locus single nucleotide polymorphism (SNP, rs35349669) of *INPP5D* which was significantly associated with susceptibility to late-onset Alzheimer's disease (LOAD) in Caucasians. In this study, we investigated the relations between the *INPP5D* polymorphism rs35349669 and LOAD in Han Chinese population comprising 984 LOAD cases and 1352 healthy controls being matched for age and gender. Our results showed no obvious differences in the genotypic or allelic distributions of rs35349669 polymorphism between LOAD cases and healthy controls (genotype: *p* = 0.167; allele: *p* = 0.094). Additionally, when these data were stratified by *APOE*ε4 status, there are still no evident differences in the genotypic or allelic distributions in *APOEε4* carriers (*p* > 0.05). Furthermore, meta-analysis of 81964 individuals confirmed that rs35349669 was significantly associated with the risk for LOAD (OR=1.08, 95%CI=1.06-1.11), but the results remained negative in Chinese subgroup (OR=0.77, 95%CI=0.53-1.13). Overall, the current evidence did not indicate that *INPP5D* rs35349669 polymorphism play a role in the genetic predisposition to LOAD in Chinese population.

## INTRODUCTION

Alzheimer's disease (AD), the most common form of dementia in the elderly [1], is a neurodegenerative disorder that is pathologically characterized by deposition of extracellular amyloid-beta (Aβ) plaques, intracellular neurofibrillary tangle composed of hyperphosphorylated tau and massive neuronal loss [2, 3]. It is well acknowledged that genetic variations play an important role in the progression of AD [4]. Mutations in *APP*, *PSEN1* and *PSEN2* are associated with early-onset familial AD, which is responsible for part of AD [5]. The genetic background of the more common late-onset Alzheimer's disease (LOAD) is more complicated, and only the variant of the apolipoprotein E (*APOE*) gene has been consistently related to the risk of this disease [6]. In addition to the established association of *APOE*, recent genome-wide association studies (GWAS) have identified other 20 genes/loci that affected the susceptibility of LOAD [7].

Given the significant part of inflammation in AD pathogenesis, inflammation-relevant genes including Inositol polyphosphate-5-phosphatase (*INPP5D)* are increasingly investigated in AD genetic association studies. *INPP5D*, mainly expressed in hematopoietic cells, plays an important role in a series of inflammatory responses [8].It has also reported that *INPP5D* was implicated in the pathogenesis of LOAD through the regulation of microglial cell function [8, 9].Recently, *INPP5D* rs35349669 polymorphism was recognized to be strongly associated with the development of LOAD in 74,046 Caucasians [10]. Since variants and their frequencies of *INPP5D* in diverse ethnic groups might be different, replication is necessary to validate the potential effects of *INPP5D* in non-Caucasian population including Asians. To address this question, the present study was conducted to evaluate the association of rs35349669 SNP with LOAD in Northern Han Chinese population.

## RESULTS

The demographic and clinical characteristics of the subjects are detailed in Table 1. LOAD patients were well-matched with controls in terms of age and gender. No statistically significant differences were detected for age (age at onset for LOAD and age at examination for controls) and gender (*P* > 0.05). As expected, MMSE scores were obviously less in LOAD patients than that in control subjects (*P* < 0.001). The *APOE4* allele frequency was also obviously different between patients and control subjects (*P* < 0.001). Distributions of the *APOE* and rs35349669 genotypes in cases and controls were all in the Hardy–Weinberg equilibrium (*P* > 0.05).

**Table 1:** The characteristics of the study population

	AD (*n* = 984)	HC (*n* = 1352)	*P* value
Age, years; mean±SD	75.15±6.08	75.50±6.49	0.185*
Gender, n (%)			0.074
Male	406 (41.3)	608 (45.0)	
Female	578 (58.7)	744 (55.0)	
MMSE score, mean±SD	11.99±6.20	28.49±1.09	<0.001
APOE ε4 status, *n* (%)			<0.001
APOE ε4 (+)	280 (28.5)	191 (14.1)	
APOE ε4 (-)	704 (71.5)	1161 (85.9)	

Abbreviations: AD, Alzheimer's disease; HC, healthy controls; MMSE, Mini-Mental State Examination; *ApoE*; apolipoprotein E; SD, standard deviation.

* *P* value was calculated with the age of onset for late-onset AD and age at examination for control. Differences in the characteristics of age and MMSE score between the two groups were examined using Student's t test. Differences in gender and ApoE-ε4 frequency between AD patients and HC were assessed using the Pearson χ2 test.

**Figure 1:**
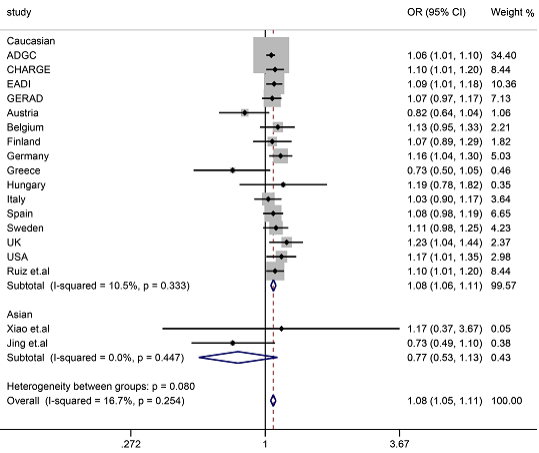
Forest plot for rs35349669 in LOAD and healthy controls in 81964 individuals

The allele and genotype distributions of rs35349669 in two groups are presented in Table 2. The genotype and allele frequencies did not differ between LOAD and controls (genotype *P* = 0.167, allele *P* = 0.094). To rule out blending effects in our initial association analyses, we reassessed rs35349669 effects under various genetic models in logistic regression adjusting for age at onset in LOAD patients (age at examination in control group), gender, and *APOE* ε4 status. Disappointedly, the result also failed to reveal any significant difference between LOAD and controls. Thus, our study observed no significant differences in the genotypic or allelic distributions of rs35349669 polymorphism between LOAD cases and healthy controls in a Northern Han Chinese population. Meanwhile, we carried out a meta-analysis about the association of rs35349669 with LOAD and found rs35349669 was strongly associated with LOAD (OR = 1.08, 95%CI = 1.05-1.11) (Figure 1) without evident analysis heterogeneity (I^2^ = 16.7%). In subgroup analysis, the rs35349669 polymorphism was also significantly associated with the risk for LOAD in Caucasian, however, the results showed that there was no association of rs35349669 with AD risk in Chinese population (OR = 0.77, 95%CI = 0.53~1.13).

**Table 2:** Distribution of the rs35349669 genotypes and alleles in the entire group and subgroup stratified by *APOE ε4*

rs35349669	*n*	Genotypes*n* (%)				Allele*n* (%)		
		TT	CT	CC	*P*	T	C	*P*
AD	984	0 (0)	54 (5.5)	930 (94.5)	0.167	54(2.7)	1914 (97.3)	0.094
Controls	1352	2 (0.1)	94 (7.0)	1256 (92.9)		98(3.6)	2606 (96.4)	
*APOE ε4 (+)*								
AD	280	0 (0)	18 (6.4)	262 (93.6)	0.713	18 (3.2)	542 (96.8)	0.708
Controls	191	0 (0)	14 (7.3)	177 (92.7)		14 (3.7)	368 (96.3)	
*APOE ε4 (-)*								
AD	704	0 (0)	36 (5.1)	668 (94.9)	0.176	36 (2.6)	1372 (97.4)	0.075
Controls	1161	2 (0.2)	80 (6.9)	1079 (92.9)		84 (3.6)	2238 (96.4)	

In addition, we assessed the influence of the interaction between rs35349669 and *APOE* genotype on the risk for LOAD in logistic regression models (Table 3), no interaction on AD risk was detected here (dominant, *P* = 0.151; additive, *P* = 0.127). In order to further investigate whether the presence of the *APOE* ε4 allele modified the association of rs35349669 with LOAD, the total group was stratified in by *APOE* ε4 carriers and *APOE* ε4 noncarriers. We observed no significant differences between AD and controls in genotype and allele distributions (*P* > 0.05) (Table 2), and in the multivariate analysis (Table 3). To exclude the effects of gender on our initial association analyses, the total group was also stratified into male and female subgroup by gender (Table 4). Likewise, the frequency of genotypes and allele for rs35349669 did not show any obvious differences between AD and controls in male or female subgroup (*P* > 0.05) (Table 4).

**Table 3:** Logistic regression analysis of INPP5D rs35349669 and AD

SNP	Total sample^a^			*APOE* ε4 (+)^b^		*APOE*ε4 (-)^b^	
rs35349669	OR(95%CI)	*P*	*P* for APOE interaction	OR(95%CI)	*P*	OR(95%CI)	*P*
Dom	0.773 (0.543-1.099)	0.151	1.167	0.846 (0.407-1.758)	0.654	0.744 (0.495-1.117)	0.154
Add	0.763 (0.539-1.080)	0.127	0.689	0.846 (0.407-1.758)	0.654	0.733 (0.492-1.093)	0.128
Rec	-	-	-	-	-	-	-

*APOE, apolipoprotein E*; *ApoE*ε4 (+), *APOE* ε4 allele carrier; *ApoEε4* (−), *APOE ε4* allele noncarrier; SNP, single nucleotide polymorphism; Dom, dominant; Rec, recessive; Add, additive model; OR, odd ratio; CI, confidence interval

a , adjusted for age, gender, and *APOE* ε4 allele status;

b , adjusted for age, gender.

- , the recessive genetic model could not be analysed when minor allele homozygote counts <14.

**Table 4:** *INPP5D* genotype and allele frequencies in AD cases and controls stratified by sex

	*N*	Genotypes, *n* (%)			*P*	Alleles, *n* (%)		*P*
		CC	TC	TT		C	T	
AD	984	930 (94.5)	54 (5.5)	0(0)	0.167	1914 (97.3)	54 (2.7)	0.094
Controls	1352	1256 (92.9)	94 (7.0)	2 (0.1)		2606 (96.4)	98 (3.6)	
Male	1014	934 (92.11)	78 (7.69)	2 (0.20)		1946 (95.96)	82 (4.04)	
AD	406	378 (93.10)	28 (6.90)	0	0.464^a^	784 (40.29)	28 (34.15)	0.266
Controls	608	556 (91.45)	50 (8.22)	2 (0.33)		1162 (59.71)	54 (65.85)	
Female	1322	1252 (94.70)	70 (5.30)	0		2574 (97.35)	70 (2.65)	
AD	578	552 (95.5)	26 (4.50)	0	0.268^a^	1130 (43.90)	26 (37.14)	0.261
Controls	744	700 (94.10)	44 (5.9)	0		1444 (56.10)	44 (62.86)	

a Fisher's exact test was performed

## DISCUSSION

*INPP5D*, as a member of the inositol polyphosphate-5-phosphatase (INPP5) family, is located on chromosome 2q37.1 [8, 11], it was implicated in AD pathogenesis through microglia-mediated inflammatory process and immune response [8, 12, 13]. Our study replicated the association between the rs35349669 polymorphism within *INPP5D* and LOAD risk in Northern Han Chinese. Finally, we failed to find significant differences in the genotypic or allelic distributions of rs35349669 polymorphism between LOAD cases and healthy controls in a Han Chinese population, even after adjustment for age, gender, and *APOE* ε4 status, these findings might have resulted from the relatively small sample size of our experimental study, in order to reduce the possibility, we explored the association of rs35349669 with LOAD risk in the meta-analysis in 81 964 individuals including Caucasian and Chinese, rs35349669 polymorphism was strongly associated with AD risk in the entire population (OR = 1.08, 95%CI = 1.06-1.11)and in Caucasian (OR = 1.06, 95%CI = 1.01-1.10), while rs35349669 polymorphism did not relate to LOAD in Chinese. Therefore, the current evidence did not support the correlation between rs35349669 and LOAD in Chinese.

Recently, *INPP5D* rs35349669 polymorphism was identified to be strongly associated with the development of LOAD (*P* < 0.05) in two-stage meta-analysis of GWAS in 74,046 Caucasians [10]. Subsequently, Ruiz et al. confirmed the significant association in Spanish, however, our study and Xiao et al. failed to replicate the association in Caucasian population. Several factors may be responsible for the inconsistency: Firstly, the genetic heterogeneity which is inherent in different ethnic populations could be the main reason. The minor allele frequency (MAF) of rs35349669 in Caucasians is significantly different from the MAF of rs35349669 in Chinese based on the dbSNP database (http://www.ncbi.nlm.nih.gov/projects/SNP). Alternatively, the effects of rs35349669 may be population-specific, possibly due to specific interactions between gene and environment. The differentiated effects of *INPP5D* rs35349669 polymorphism on AD probably attribute to a brain structural mechanism for such population specific genetic effects. In addition to the population specification and special genetic backgrounds, environments, educational background, sample size, etc. may be the source of different results between Caucasian and Chinese. On the other hand, although our study failed to replicate any association of the examined SNP with LOAD in Chinese, we could not rule out the possibility that other SNPs of *INPP5D* associated with LOAD.

The function of *INPP5D* in immune response and inflammation in the central nervous system(CNS) is still poorly understood. Recent studies showed that the human SHIP protein, encoded by the *INPP5D* gene, was supposed to restrain the release of diverse inflammatory cytokines from microglia,astrocytes or even neurons, such as IL-6, IL-8,TNF-α [11]. In addition, a previous study revealed that the binding of *INPP5D* with the product of *CD2AP*, another LOAD risk gene, could control degradation of IgE receptor FceRIc, some IgE receptors are members of the *MS4A* gene superfamily that has been correlated with LOAD risk [14]. Besides, according to a clinical-pathologic correlation study about AD, the minor allele (T) from rs35349669 of the *INPP5D* gene was associated with other neuropathologies coexist with AD pathology, such as microinfarcts and LB disease [15]. With renewed genetic sequencing about *INPP5D* in the near future, it may be helpful to find new loci that related to LOAD.

In conclusion, we were not able to detect the significant association of *INPP5D* rs35349669 polymorphism with LOAD in Chinese. It is likely that the implication of *INPP5D* variation in AD risk may be specific to particular ethnic groups, besides, the implication is too small to be detected responsibly by a cohort of our size. It warranted investigators to clarify the role of *INPP5D* polymorphisms in LOAD in larger cohorts and in other ethnic populations.

## MATERIALS AND METHODS

### Subjects

We investigated 984 sporadic LOAD patients (mean age at onset: 75.15 ± 6.08 years; 578women) and 1352 healthy control subjects (mean age at examination: 75.50 ± 6.49 years; 744 women) matched for sex and age. All the subjects were unrelated Northern Han Chinese originated from Shandong Province. The patients were recruited from the Department of Neurology at Qingdao Municipal Hospital and some other hospitals in Shandong province. The diagnose of probable AD met with the criteria of the National Institute of Neurological and Communicative Disorders and Stroke and the Alzheimer's disease and Related Disorders Association [16]. No family history of neurodegenerative disorders or other dementias were reported among AD patients. The control subjects were enrolled from the Health Examination Center of the Qingdao Municipal Hospital according to the principles described elsewhere [2].They were identified healthy and neurologically normal according to medical history, general examinations, laboratory examinations and acquired ≥28 points on the Mini-Mental State Examination (MMSE). Informed consent was obtained from all individuals or from the subject's guardian, and the protocol of this study was approved by the Ethical Committee of Qingdao Municipal Hospital.

### Genotyping

Genomic DNA was extracted from peripheral blood leukocytes of AD patients and healthy individuals using the Wizard genomic DNA purification kit (Cat. #A1125, Promega, USA).Genotyping of *INPP5D* (rs35349669) and *APOE* polymorphisms was performed by a custom-by-design 48-PlexSNP scan TM Kit (Genesky Biotechnologies, Inc., Shanghai, China), as previously described [17]. This was high-throughput and cost-saving SNP genotyping methods based on double ligation and multiplex fluorescence PCR. The genotyping of *APOE* were carried out by Shanghai Genesky Bio-Tech Co., Ltd. (http://biotech.geneskies.com/index.html) using the improved multiplex ligase detection reaction (iMLDR) method [18]. Data analysis was accomplished using GeneMapper Software v4.0 (Applied Biosystems). Randomly selected DNA samples from each genotype were analyzed in duplicate using ligation detection reaction and sequence analysis method. Consistent results were achieved by these two methods.

### Statistical analysis

Statistical analysis was performed using SPSS16.0 software. Genotype and allele frequencies were calculated by counting. The Hardy–Weinberg equilibrium (HWE) was tested for each SNP with genotype data from both AD patients and controls. Differences in the characteristics between the two groups were tested using Student's t-test or the chi-square test. Genotypes and alleles frequencies between LOAD and healthy control group were compared using Pearson χ2 test or Fisher's exact test. Differences between cases and controls after stratification for *APOE4* status were also examined by the chi-square test. The association of rs35349669 with LOAD risk was further analyzed using logistic regression adjusting for age, gender, and *APOE 4* status under various genetic models that were defined as 1 (TT + TC) versus 0 (CC) for dominant, 1 (TT) versus 0 (TC + CC) for recessive, and 0 (CC) versus 1 (TC) versus 2 (TT) for additive. The P value, odds ratios (ORs) and 95% confidence intervals (CIs) were calculated. Estimation of the statistical power was performed with the STPLAN 4.3 software. Values of *P* < 0.05 were considered statistically significant.

Additionally, we also combined our data with the results from other studies about rs35349669 and LOAD risk [10, 19, 20] through fixed-effects inverse variance-weighted methods. Meanwhile, we generated I^2^ estimates with evaluate the possible effect of study heterogeneity on the results. Stata V.12.0 was used to perform all these analyses.
